# Performance and return to sport outcomes following hip arthroscopy in National Hockey League players

**DOI:** 10.1002/ksa.12720

**Published:** 2025-06-12

**Authors:** David Slawaska‐Eng, Marc Daniel Bouchard, Luigi Del Sordo, Alexander E. Weber, Olufemi Ayeni

**Affiliations:** ^1^ Division of Orthopaedic Surgery, Department of Surgery McMaster University Hamilton Ontario Canada; ^2^ Michael G. DeGroote School of Medicine McMaster University Hamilton Ontario Canada; ^3^ Division of Orthopaedic Surgery, Keck School of Medicine University Southern California Los Angeles California USA

**Keywords:** arthroscopy, FAIS, ice hockey, National Hockey League, labral tear

## Abstract

**Purpose:**

Intra‐articular hip disorders, such as femoroacetabular impingement syndrome (FAIS), labral tears and chondral damage are common in ice hockey players, particularly in the National Hockey League (NHL). However, evidence on return‐to‐sport (RTS) rates and performance outcomes post‐hip arthroscopy remains limited. This study evaluates RTS rates, career longevity, and performance metrics, including games played, points per game (PPG), save percentage, and performance scores (PS), following hip arthroscopy.

**Methods:**

NHL players who underwent hip arthroscopy for intra‐articular pathology between 2000 and 2024 were identified using public records. RTS rates, career duration, and performance metrics were analysed pre‐ and post‐surgery. Paired *t*‐tests and analyses of variance (ANOVA) were performed across positions (forwards, defensemen and goaltenders).

**Results:**

A total of 92 NHL players (103 hips) met inclusion criteria. The overall RTS rate was 79.3%, increasing to 84.9% when excluding players still recovering. RTS was significantly higher in players <30 years (90.0% vs. 64.3%, *p* = 0.003). The average number of post‐operative seasons played was 2.7, with no positional differences. Forwards showed significant declines in PPG (pre: 0.63 ± 0.38; post: 0.51 ± 0.37; *p* = 0.013) and PS (pre: 0.60 ± 0.74; post: 0.37 ± 0.69; ∆PS = −0.23; *p* = 0.026). Defensemen showed no significant change in PPG (*p* = 0.648) or PS (*p* = 0.509). Goaltenders had a decline in save percentage (pre: 0.91 ± 0.01; post: 0.89 ± 0.03; *p* = 0.038), while wins per season were unchanged (*p* = 0.205). RTS did not significantly differ by position.

**Conclusion:**

NHL players undergoing hip arthroscopy have high RTS rates and often resume multi‐season careers. However, forwards experience greater declines in performance, while defensemen and goaltenders are less affected. These results underscore position‐specific recovery trends and may inform rehabilitation strategies in elite hockey athletes.

**Level of Evidence:**

Level V.

**Abbreviations**
ANOVA: analysis of variance
ATOI: average time on ice
CI: confidence interval
ESG: even‐strength goals
FAIS: femoroacetabular impingement syndrome
GP: games played
GWG: game‐winning goals
NHL: National Hockey League
PIM: penalties in minutes
PPG: points per game
PS: performance score
PWPG: power play goals
RTS: return to sport
SV%: save percentage
WPS: wins per season

## INTRODUCTION

Ice hockey is a highly physical sport that demands a combination of speed, teamwork and power [17]. Its increasing global popularity has highlighted the substantial physical demands placed on players, particularly at elite levels. The sport's high‐speed nature and intense physicality exposes players to a wide range of potential injuries, with the risk escalating as players become more powerful and faster [1, 19]. Research indicates that athletes in higher‐calibre leagues, such as the National Hockey League (NHL), experience injuries more frequently than their counterparts in lower‐tier leagues [17]. Given the predominantly lower‐extremity biomechanical demands of ice hockey, hip injuries are among the most frequently sustained musculoskeletal injuries in this population [1].

In the NHL, approximately 10%–12% of all hip and groin injuries are intra‐articular, placing players at increased susceptibility to conditions such as hip labral tears, femoroacetabular impingement syndrome (FAIS), chondral damage, and loose bodies [23]. FAIS arises from abnormal morphology in the hip joint, which can occur on the femoral side (cam‐type morphology) or acetabular side (pincer‐type morphology) [21]. This morphological abnormality results in pathologic contact and mechanical forces across the joint, contributing to labral and chondral pathologies [21]. Due to the highly repetitive flexion and internal rotation motions required in ice hockey, cam morphology is more common and players tend to present with activity‐related groin or hip pain [9]. Players frequently report mechanical symptoms, including locking, clicking or catching, as well as restricted range of motion, which can adversely affect their performance [21]. Some literature would suggest that goaltenders are at a greater risk of an intra‐articular hip injury than other players when measured per game played [5].

Hip arthroscopy is the preferred treatment option for FAIS, aiming to correct joint morphology and address associated pathologies [9, 21]. By alleviating pain and improving range of motion, it facilitates an athlete's return to sport (RTS). Previous studies have reported RTS rates following hip arthroscopy for FAIS ranging from 78% to 100%, but the definition of RTS varies across studies and does not always reflect a player's ability to return to preinjury performance levels [9, 11, 14, 20]. Furthermore, there is a notable lack of evidence evaluating performance‐based outcomes exclusively among NHL players following hip arthroscopy.

The purpose of this study is to determine the RTS rate and performance‐based outcomes specifically among professional NHL players following hip arthroscopy. By utilising standardised RTS definitions and analysing the largest sample of professional hockey players to date, this study hypothesises that a high proportion of NHL players will successfully return to play following arthroscopic treatment for FAIS and labral tears. Additionally, we aim to compare RTS outcomes across different player positions to identify potential positional variations in recovery and performance metrics.

## METHODS

By using previously published methodologies [7, 10, 16, 18], we identified NHL players who sustained intra‐articular hip injuries requiring arthroscopic management between the 2000–2001 and 2023–2024 seasons from the publicly available NHL Injury Viz database [24]. To ensure completeness and account for potential misclassification of hip injuries, all players with injuries classified as 'hip', 'lower body' and 'groin' were extracted. Players who underwent hip arthroscopy were confirmed through at least two publicly available sports reporting websites, including TSN.ca, ESPN, and FoxSports.com.

Players were included in the study if they were placed on the injured list and had their hip arthroscopy corroborated by at least two independent public sources. To minimise confounding variables, players with concomitant injuries requiring surgery were excluded. Additionally, players who did not return to sport were included in RTS analyses but excluded from performance variable analyses.

### Demographic and injury variables

Data collected for each player included age at time of injury, height, weight, position, games missed, diagnosis subtype, laterality of injury (unilateral vs. bilateral) and years of NHL experience at the time of injury.

### Data abstraction

For eligible players, all regular‐season game statistics were retrieved using Hockey‐Reference.com [25], covering data from their rookie year through the 2023–2024 seasons. To ensure data reliability, a sample extraction of 10 players' statistics was conducted by two independent reviewers and verified by a senior author before proceeding with full data extraction.

Variables extracted for forwards and defensemen included games played (GP), goals, assists, points, plus/minus rating, penalties in minutes (PIM), even‐strength goals (ESG), power play goals (PWPG), game‐winning goals (GWG), shots on goal (SOG) and average time on ice (ATOI). For goalies, variables included GP, wins per season (WPS), losses, ties plus overtime losses, shutouts, saves, goals against, total game minutes and save percentage (SV%). Pre‐ and post‐operative statistics were obtained. The post‐operative period was defined by the first full season following surgery.

### Outcome measures

While RTS was defined as return to play for ≥1 NHL regular‐season game, functional performance was evaluated separately using metrics such as games played per season, PPG, ATOI, and a validated hockey‐specific performance score (PS) as described by Schroeder et al. [16] (Table 1). Return to pre‐injury performance was assessed using pre‐ and post‐operative PS. For goalies, outcome measures included save percentage and wins per season (WPS).

**Table 1:** Performance score formula for a given position.

Forward	$\frac{\left(3.0\times \mathrm{ESG})+\left(2.0\times \mathrm{PWPG}\right)+(4.0\times \mathrm{SHG})+(4.0\times \mathrm{GWG})+\left(2.0\times \mathrm{assists}\right)+(\mathrm{plus}/\mathrm{minus})-\left(0.25\times \mathrm{PIM}\right)-(0.33\times \mathrm{SOG}\right)}{\mathrm{Games}\;\mathrm{Played}}$
Defenseman	$\frac{\left(5.0\times \mathrm{ESG})+\left(4.0\times \mathrm{PWPG}\right)+(6.0\times \mathrm{SHG})+(5.0\times \mathrm{GWG})+\left(3.0\times \mathrm{assists}\right)+(\mathrm{plus}/\mathrm{minus})-\left(0.25\times \mathrm{PIM}\right)-(0.33\times \mathrm{SOG}\right)}{\mathrm{Games}\;\mathrm{Played}}$
Goalie	$\frac{\left(0.7\times \mathrm{wins}\right)+\left(0.2\times \mathrm{ties}+\mathrm{over}\;\mathrm{time}\;\mathrm{losses}\right)+\mathrm{shutouts}+\left(0.17\times \mathrm{saves}\right)-\left(0.25\times \mathrm{losses}\right)-\left(1.23\times \mathrm{goals}\;\mathrm{against}\right)}{\mathrm{Games}\;\mathrm{Played}}$

Abbreviations: ESG, even strength goals; GWG, game‐winning goals; PIM, penalty in minutes; PWPG, power play goals; SHG, short‐handed strength goals; SOG, shots on goal.

### Statistical analysis

Pre‐ and post‐operative statistics were calculated with post‐operative performance defined by the sum of the season statistics beginning from the first full season following surgery. As no validated PS cutoff has been established, changes were evaluated based on statistical significance rather than a fixed threshold. Continuous pre‐ and post‐operative variables were compared within each cohort using paired *t*‐tests. Two‐proportion z‐test or Chi‐square test were used for dichotomous variables. Analysis of variance (ANOVA) was used to detect differences in pre‐ and post‐operative PS between forwards, defensemen, and goalies. A post hoc power analysis was performed on all statistically significant outcomes where high post hoc power is defined as >0.8. For all analyses, statistical significance was set at *p* < 0.05. All statistical analyses were performed using Microsoft Excel.

## RESULTS

### Study sample

**Figure 1:**
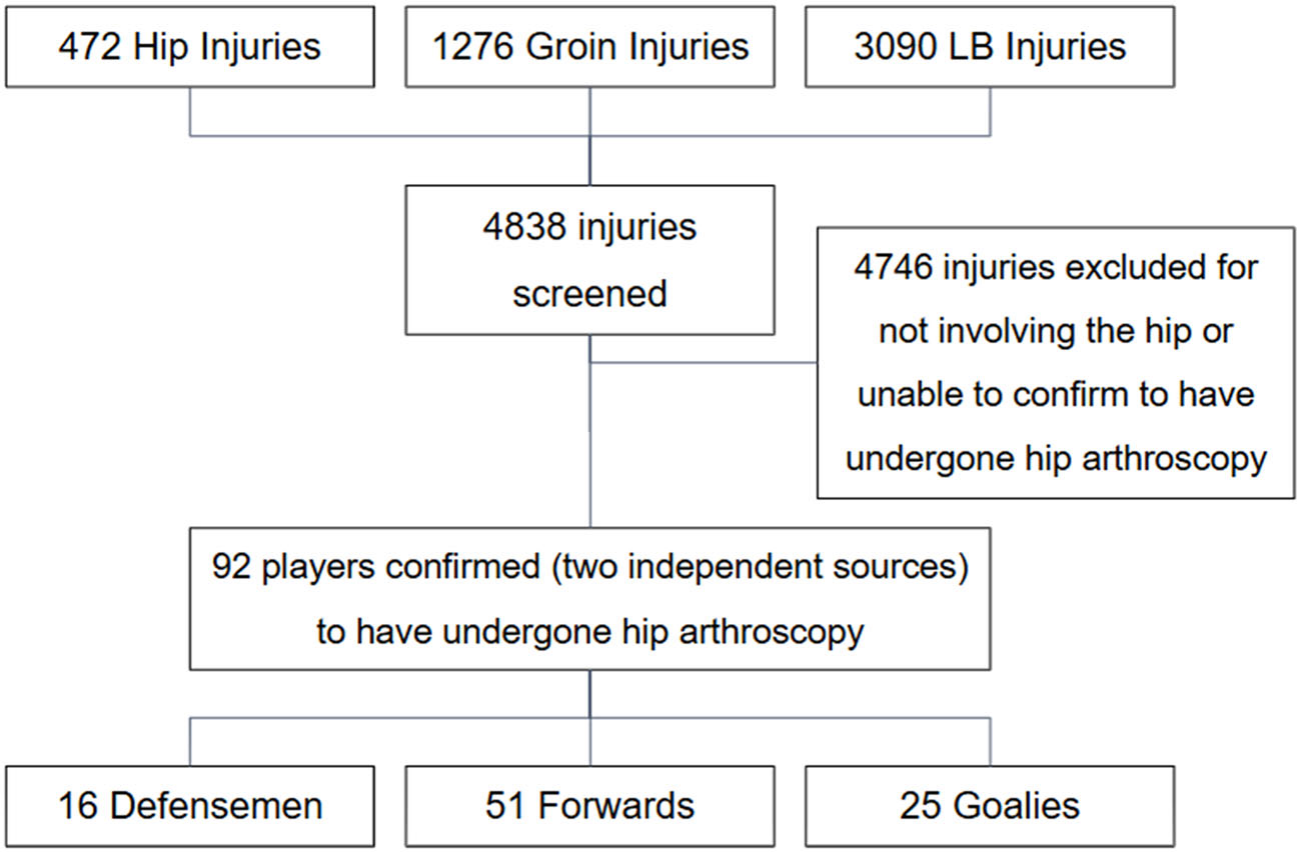
Flowchart of participants. Injuries were obtained the hip, groin and lower body (LB) Injury Viz Databases.

The final sample consisted of 92 NHL players who underwent arthroscopic hip surgery. This sample was derived from an initial screening of 4838 player injuries (472 hip injuries, 1276 groin injuries and 3090 lower body injuries), of which 4735 were excluded due to being unrelated to the hip or not involving hip arthroscopy (Figure 1). Among the included players, 53 were diagnosed with labral tears, eight with FAIS, and 25 with non‐specific hip injuries but confirmed to have undergone hip arthroscopy. Additional diagnoses included arthritis (*n* = 2), cartilage tears (*n* = 2), bacterial infections (*n* = 1) and loose bodies requiring removal (*n* = 1). Of the 92 players, 11 had bilateral injuries, yielding a total of 103 hips analysed. The sample comprised 16 defensemen, 51 forwards and 25 goalies, with a mean age of 28.9 ± 3.9 years (range: 21–38) at the time of diagnosis (Table 2).

**Table 2:** Demographics of included NHL players.

	All players	Defenseman	Forward	Goalie
*n*	92	16	51	25
*n* RTS	73	12	46	19
% RTS	79.4%	75.0%	90.2%	76.0%
Games missed (SD)	28.3 (24.9)	32.8 (20.9)	26.0 (24.7)	30.1 (27.9)
Bilateral injury (*n*)	11	1	5	5
% Bilateral	12.0%	6.3%	9.8%	20.0%
Age at time of diagnosis (SD)	28.9 (3.9)	28.3 (3.9)	29.0 (4.4)	29.0 (3.0)
Height [in] (SD)	73.2 (2.0)	73.6 (2.0)	72.7 (1.8)	73.8 (2.2)
Weight [lbs] (SD)	202.1 (17.0)	212.7 (14.2)	196.3 (17.6)	199.8 (12.4)
Years of NHL experience at time of injury (SD)	8.4 (4.1)	7.5 (3.5)	8.9 (4.4)	7.9 (3.6)

Abbreviations: NHL, National Hockey League; RTS, return to sport; SD, standard deviation.

### RTS

The overall RTS rate for NHL players following hip arthroscopy was 79.3% (73 of 92 players). RTS rates by position were as follows: forwards—82.4% (42/51), defensemen—75.0% (12/16) and goalies—76.0% (19/25). A chi‐square test of independence showed no significant difference in RTS rates between positions (*χ*² = 0.64, *p* = 0.73). RTS rates were stratified by age and were significantly higher in players <30 years of age compared to those ≥30 years (90.0% [45/50] vs. 64.3% [27/42]; *z* = 2.98, *p* = 0.003). The mean number of seasons played for all players post‐injury is 2.7 ± 2.2. There was no statistically significant difference in the number of post‐injury seasons played among forwards, defensemen, and goalies (2.7 ± 2.3 vs. 3.1 ± 2.2 vs. 2.4 ± 1.9; *p* = 0.69).

Players who did not return included those who retired immediately after surgery (*n* = 13; one defenseman, nine forwards and three goalies) or were still considered to be active (non‐retired) in the league and recovering from surgery (*n* = 6; three defensemen, one forward and two goalies). When removing these six players from the calculation and accounting for only those who had retired from the NHL following their surgery, the RTS rate increased to 84.9% (73 of 86 players).

### PS

**Figure 2:**
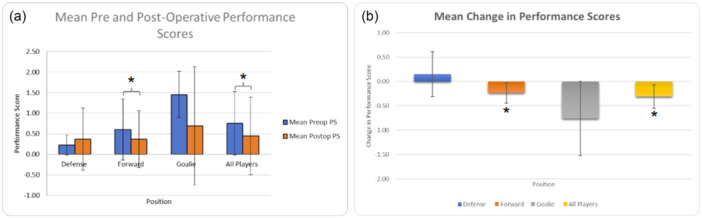
(a) Mean pre‐ and post‐operative performance scores (PS) in defensemen (*n* = 12), forwards (*n* = 42), goalies (*n* = 19) and all players pooled together (*n* = 73). Error bars represent the standard deviation (SD). (b) Change in mean PS in defensemen, forwards goalies and all players pooled. Error bars represent the 95% confidence interval (95% CI). The * indicates statistical significance (*p* < 0.05).

Pre‐ and post‐operative PS were analysed for defensemen, forwards, goalies and all players combined. Declines in PS were observed across all groups, with statistically significant reductions for forwards (pre‐operative PS: 0.60 ± 0.74; post‐operative PS: 0.37 ± 0.69; ∆PS = −0.23, 95% confidence interval [CI] [−0.44; −0.03]; *p* = 0.026) and all players combined (pre‐operative PS: 0.77 ± 0.77; post‐operative PS: 0.46 ± 0.95; ∆PS = −0.31, 95% CI [−0.55; −0.07]; *p* = 0.039). No statistically significant changes were observed for defensemen (pre‐operative PS: 0.25 ± 0.24; post‐operative PS: 0.40 ± 0.78; ∆PS = 0.15, 95% CI [−0.34; 0.64]; *p* = 0.509) or goalies (pre‐operative PS: 1.45 ± 0.56; post‐operative PS: 0.69 ± 1.43; ∆PS = −0.76, 95% CI [−1.52; 0]; *p* = 0.051) (Figure 2a,b). The ANOVA revealed no significant differences in mean ∆PS among forwards, defensemen, and goalies (*p* > 0.05).

### Career statistics (points per game [PPG], ATOI and GP)

Comparisons of career statistics pre‐ and post‐surgery revealed a statistically significant reduction in mean PPG for forwards (pre‐operative PPG: 0.63 ± 0.38; post‐operative PPG: 0.51 ± 0.37; ∆PPG = −0.11, 95% CI [−0.20; −0.03]; *p* = 0.013), but not for defensemen (pre‐operative PPG: 0.23 ± 0.07; post‐operative PPG: 0.22 ± 0.12; ∆PPG = −0.02, 95% CI [−0.09; 0.06]; *p* = 0.648).

Forwards also experienced a statistically significant reduction in ATOI post‐surgery (pre‐operative ATOI: 15.41 ± 3.88; post‐operative ATOI: 14.43 ± 4.00; ∆ATOI = −0.98, 95% CI [−1.81; −0.14]; *p* = 0.023). There was a slight but not statistically significant decrease in mean average ATOI for defenseman pre‐operatively compared to post‐operative (pre‐operative ATOI: 18.55 ± 2.77; post‐operative ATOI: 17.40 ± 2.45; ∆ATOI = −1.15, 95% CI [−3.24; 0.94]; *p* = 0.251).

The mean games played per season post‐surgery were 47.04 ± 15.34 for defensemen, 55.97 ± 18.75 for forwards and 29.33 ± 14.26 for goalies. These values were not significantly different from the pre‐operative games played per season for any position (*p* > 0.05).

### Goalie‐specific statistics

For goalie‐specific metrics, there was a statistically significant reduction in save percentage (pre‐operative SV%: 0.91 ± 0.01; post‐operative SV%: 0.89 ± 0.03; ∆SV% = −0.02, 95% CI [−0.04; 0]; *p* = 0.038), while no statistically significant change was detected in wins per season between the pre‐operative and post‐operative groups (pre‐operative WPS: 12.73 ± 7.26; post‐operative WPS: 10.11 ± 8.61; ∆WPS = −2.62, 95% CI [−8.23; 3.00]; *p* = 0.205).

### Post hoc power analysis

A post hoc power analysis was conducted to determine whether the study was adequately powered to detect significant differences in performance outcomes. The study had moderate power (0.78) to detect changes in overall PS among all players. The study was underpowered for position‐specific comparisons: forwards' PS (power = 0.34), forwards' PPG (0.31) and forwards' ATOI (0.24). In contrast, the study was fully powered (1.00) to detect the significant reduction in goaltender SV%.

## DISCUSSION

Ice hockey demands exceptional coordination, balance and strength, placing substantial stress on the lower extremities, particularly the hip joint [13, 22]. Intra‐articular hip disorders, such as labral tears and FAIS, are significant contributors to morbidity among NHL players, frequently necessitating arthroscopic intervention [13]. Our study found a RTS rate of 79.3% following hip arthroscopy, which increased to 84.9% when excluding active players still recovering from surgery. This adjusted rate reflects that RTS is a dynamic process, where some athletes may return in subsequent seasons, a pattern also observed in other professional sports. Prior studies with long‐term follow‐up have shown that RTS rates may continue to increase up to two years postoperatively, suggesting that short‐term RTS rates may underestimate the full extent of recovery [15].

Compared to an earlier study by Philippon et al. in 2010 (RTS: 100%) looking at arthroscopic labral repair and treatment of femoroacetabular impingement in 28 NHL players, our findings indicate a slightly lower RTS rate [15]. Since both studies analysed NHL players exclusively, the difference in RTS rates is likely attributable to variations in study design, follow‐up duration, inclusion criteria, and patient selection. One key difference is that Philippon et al. excluded players with bilateral hip symptoms, whereas our study included them, potentially introducing a cohort with more extensive hip pathology and prolonged recovery times. Additionally, Philippon et al. examined a cohort of NHL players treated at a single high‐volume surgical centre, where rehabilitation protocols and post‐operative management may have been more standardised compared to our broader data set of players treated across multiple institutions. Their study also required a minimum of 1‐year follow‐up, whereas our methodology inherently captures some players with shorter post‐operative windows, which may lead to an underestimation of long‐term RTS rates.

Despite these differences, our RTS rate aligns closely with the 78% RTS rate reported by Lindman et al. in 2022, who examined a cohort of professional and subelite ice hockey players from an entire local Swedish hip arthroscopy registry [9]. While their study included a broader range of competitive levels, the comparable RTS rate suggests that return‐to‐sport outcomes following hip arthroscopy remain relatively consistent across different playing levels thereby, reinforcing the external validity of our findings.

Despite favourable RTS rates, post‐operative performance metrics showed variability. While players generally maintained pre‐injury levels in terms of games played per season, forwards exhibited statistically significant declines in ATOI and PPG. PS decreased across all positions, with forwards experiencing the most substantial and statistically significant decline. One likely explanation is the high biomechanical demands placed on forwards, who engage in more frequent high‐intensity skating bursts, rapid changes in direction, and greater lower‐body rotational torque, all of which could be affected by post‐surgical joint mechanics [22]. Forwards, in particular, have been shown to perform significantly more high‐intensity skating per minute than defensemen, with up to 54% greater high‐speed efforts [8]. Additionally, studies have demonstrated that forwards engage in more sprinting (>24 km/h) and very fast‐speed skating (>21 km/h) compared to defensemen, accumulating substantial time at near‐maximal skating speeds per shift [4, 6]. This heightened intensity places increased demands on acceleration, deceleration, and edge control, further stressing the hip joint. These findings suggest that rehabilitation protocols may need to be position‐specific, with particular emphasis on neuromuscular retraining for forwards, incorporating strategies to mitigate the high‐impact forces and rapid directional changes inherent in their playing style.

Goaltenders comprised 27.2% of our cohort, despite representing a smaller league‐wide proportion. This may reflect heightened positional susceptibility, as prior data show higher per‐game injury rates among goaltenders (1.84 per 1000 player‐game appearances) compared to defensemen (0.47) and forwards (0.34) [5]. The butterfly technique requires repetitive hip flexion and internal rotation, placing considerable biomechanical stress on the joint and may predispose goalies to intra‐articular injury. Despite this, goaltenders exhibited the smallest decline in PS following hip arthroscopy. This may, in part, be attributed to their unique playing schedule and recovery opportunities. Unlike skaters, who are expected to play more frequently and rely on explosive strides and high‐speed transitions, goaltenders often have designated rest days and may not be expected to play every game [13]. As a result, they may return to competition only when fully recovered, selectively participating in games when they feel at or near 100%. This ability to modulate workload postoperatively may facilitate a more effective return to pre‐injury performance levels.

While statistically significant reductions in PS were observed, the clinical significance of these declines remains uncertain. Although a decrease in PS may indicate a drop in player efficiency, it is unclear whether these changes are meaningful in terms of career sustainability. To assess this, future research should evaluate contract retention rates, post‐operative salary trends, and longevity in the NHL following hip arthroscopy. Additionally, establishing a clinically meaningful PS threshold would provide greater context for these findings.

Our findings align with existing sports medicine literature on RTS and post‐operative performance outcomes in NHL players. Schroeder et al.'s 2013 study on lumbar disc herniation in NHL players reported an 85% RTS rate and statistically significant post‐operative reductions in games played, PPG and PS, paralleling our observations following hip arthroscopy [16]. The biomechanical demands of ice hockey, particularly torsional and compressive stresses during skating, may contribute to these performance declines. Conversely, Gotlin et al.'s 2020 study on upper extremity fractures in NHL players (RTS: 98%) found no significant declines in performance one year post‐injury [7]. This contrast highlights the disproportionate reliance on lower‐extremity function in ice hockey, where hip injuries and subsequent surgeries are more likely to impact key performance indicators.

The study further underscores how intra‐articular hip injuries disproportionately affect older NHL players nearing the end of their careers. The mean age of our cohort (28.9 years) aligns closely with peak NHL scoring ages (27–28 for forwards, 28–29 for defensemen) [2] and the league's median retirement age (27 years) [3]. Our findings raise the question of whether hip arthroscopy merely coincides with a natural performance decline or if it actively accelerates career transition. Our data showed that players under 30 years of age had significantly higher RTS rates than those 30 or older (90.0% vs. 64.3%, *p* = 0.003), reinforcing the importance of age as a prognostic factor. These findings are consistent with those of Menge et al., who found that younger age at the time of surgery and shorter symptom duration were associated with longer post‐operative careers [12]. Our data also demonstrated that the mean number of post‐operative seasons played was 2.7 ± 2.2, with no statistically significant difference by position. This is lower than the 5.9‐year mean post‐operative career length reported by Menge et al. (of which 67% of players played a minimum of five seasons) [12]. One likely explanation is that our study includes active players who have not yet completed their careers, whereas Menge's analysis was conducted after retirement, capturing true career longevity. As such, our post‐operative career length may be an underestimate and will require long‐term follow‐up for validation.

Our study's strengths include the largest NHL cohort analysed to date (24 seasons), a rigorous screening process, a strict RTS definition, and a validated performance scoring system. However, key limitations exist. Since NHL injury data is not systematically recorded in a league‐wide registry, we relied on publicly available reports, introducing potential selection bias. Complication rates, revision surgeries, or long‐term re‐injury rates could not be assessed due to lack of access to individual medical records. Some players who underwent hip arthroscopy but were not placed on injured reserve may have been excluded, particularly role players and backup goaltenders, whose injuries are less publicised. Injury information in professional sports is often withheld to protect players' contractual negotiations, further contributing to reporting bias. Despite this, the wide range of pre‐injury PS (mean 0.77; range −0.71 to 3.15) suggests our cohort included players of varying skill levels, mitigating some bias. Future studies should access de‐identified team medical records for a more comprehensive dataset. Lastly, while overall performance declines were detected with moderate power (0.78), the study was underpowered for position‐specific analyses, particularly among forwards, suggesting the true impact on offensive performance may be underestimated. As such, these findings should be interpreted with caution and validated in larger cohorts. In contrast, goaltender performance changes were well‐detected, reinforcing the reliability of that finding.

## CONCLUSION

While multiple factors influence performance‐based outcomes following hip arthroscopic surgery, including contractual obligations, post‐operative rehabilitation protocols and coexisting injuries, this study suggests that a high proportion of NHL players successfully return to play. These findings provide valuable insights for players, team management, and orthopaedic surgeons, facilitating informed decision‐making regarding treatment strategies for NHL athletes with intra‐articular hip disorders.

## AUTHOR CONTRIBUTIONS

David Slawaska‐Eng conceived and designed the study, collected the data, performed the statistical analysis, and drafted the manuscript. Marc Daniel Bouchard and Luigi Del Sordo assisted with data collection and contributed to manuscript writing. Dr. Alexander Weber and Dr. Olufemi Ayeni contributed to manuscript editing and critical revisions. All authors reviewed and approved the final manuscript.

## CONFLICT OF INTEREST STATEMENT

Dr. Ayeni has receives speaker fees from Stryker Canada and research funding from the Canada Research Chair, however, none of the fees or funding were used to complete this study. Dr. Weber serves as Medical Director and Head Team Physician for the Los Angeles Kings and receives educational support from Stryker USA which was not used to complete this study. The remaining authors declare no conflict of interest.

## ETHICS STATEMENT

Ethics approval was not required for this study as all data were obtained from publicly available sources and did not involve human participants directly or identifiable private information. Patient consent was not required as only publicly available information was used. No player names or other identifying details were included in the study.

## Data Availability

The data sets analysed in this study are publicly available and can be accessed from publicly accessible sources. No new data were generated during this study. Analysed raw data from the current study are available from the corresponding author on reasonable request.
